# Retinal microvascular network geometry and cognitive abilities in community-dwelling older people: The Lothian Birth Cohort 1936 study

**DOI:** 10.1136/bjophthalmol-2016-309017

**Published:** 2017-06-22

**Authors:** Sarah McGrory, Adele M Taylor, Mirna Kirin, Janie Corley, Alison Pattie, Simon R Cox, Baljean Dhillon, Joanna M Wardlaw, Fergus N Doubal, John M Starr, Emanuele Trucco, Thomas J MacGillivray, Ian J Deary

**Affiliations:** 1 Division of Neuroimaging Sciences, Centre for Clinical Brain Sciences, University of Edinburgh, Edinburgh, UK; 2 Department of Psychology, University of Edinburgh, Edinburgh, UK; 3 Faculty of Medicine, University of Split, Split, Croatia; 4 Centre for Cognitive Ageing and Cognitive Epidemiology, University of Edinburgh, Edinburgh, UK; 5 Scottish Imaging Network: A Platform for Scientific Excellence Collaboration, Edinburgh, UK; 6 Alzheimer Scotland Dementia Research Centre, University of Edinburgh, Edinburgh, UK; 7 VAMPIRE project, Computing, School of Science and Engineering, University of Dundee, Dundee, UK; 8 Edinburgh Clinical Research Facility, University of Edinburgh, Edinburgh, UK

**Keywords:** Retina, Imaging

## Abstract

**Aim:**

To examine the relationship between retinal vascular morphology and cognitive abilities in a narrow-age cohort of community-dwelling older people.

**Methods:**

Digital retinal images taken at age ∼73 years from 683 participants of the Lothian Birth Cohort 1936 (LBC1936) were analysed with Singapore I Vessel Assessment (SIVA) software. Multiple regression models were applied to determine cross-sectional associations between retinal vascular parameters and general cognitive ability (*g*), memory, processing speed, visuospatial ability, crystallised cognitive ability and change in IQ from childhood to older age.

**Results:**

After adjustment for cognitive ability at age 11 years and cardiovascular risk factors, venular length-to-diameter ratio was nominally significantly associated with processing speed (β=−0.116, p=0.01) and *g* (β=−0.079, p=0.04). Arteriolar length-to-diameter ratio was associated with visuospatial ability (β=0.092, p=0.04). Decreased arteriolar junctional exponent deviation and increased arteriolar branching coefficient values were associated with less relative decline in IQ between childhood and older age (arteriolar junctional exponent deviation: β=−0.101, p=0.02; arteriolar branching coefficient: β=0.089, p=0.04). Data are presented as standardised β coefficients (β) reflecting change in cognitive domain score associated with an increase of 1 SD unit in retinal parameter. None of these nominally significant associations remained significant after correction for multiple statistical testing.

**Conclusions:**

Retinal parameters contributed <1% of the variance in the majority of associations observed. Whereas retinal analysis may have potential for early detection of some types of age-related cognitive decline and dementia, our results present little evidence that retinal vascular features are associated with non-pathological cognitive ageing.

## Introduction

Cerebrovascular disease is associated with lower cognitive ability in older age, and is known to contribute to the pathogenesis of dementia.^1^ Yet, research has been hampered by difficulties in directly observing and measuring the cerebral microvasculature. Due to the homology between retinal and cerebral microvasculatures,^2^ using the retina as a non-invasive ‘window’ to the cerebrovasculature could help identify early microvascular abnormalities before the appearance of clinically overt disease.

Previous studies have investigated associations between retinal parameters and cognitive abilities^3–6^ and dementia.^7–9^ A systematic review of six studies^10^ found that retinopathy and retinal branching parameters were most consistently associated with poorer verbal memory, information processing speed and executive function in middle-aged and older people. Two studies of healthy older adults (n=321–2211) found small effect sizes (η^2^=0.026 to 0.037)^4^ ^11^ compared with studies of younger adults (η^2^=0.07 to 0.22).^12^ Similarly, a systematic review examining associations between microvascular changes, dementia, cognitive functioning and brain imaging abnormalities found stronger associations between retinal differences and cognitive function in early and mid-adulthood than in older age,^13^ echoing age-related weakening of associations between cardiovascular and cerebrovascular disease and retinopathy.^14^ To date, retinal differences appear to be weak indicators within the range of non-pathological age-related cognitive decline (ie, healthy/normal age-related cognitive ageing, not due to dementia or other pathological cognitive disorders).

Childhood IQ accounts for about half of the variance in cognitive status in later life.^15^ Consequently, cross-sectional associations could be confounded by prior cognitive ability. Access to a valid measure of premorbid cognitive ability is rare, and results from the few studies which have examined its contribution are inconclusive. Shalev *et al*^16^ found that wider venular width at 38 years was significantly associated with lower cognitive ability measured concurrently and in childhood, indicating that retinal parameters may reflect cognitive status years before cognitive impairment and dementia onset. Retinal branching parameters accounted for 3.7%, at most, of the variance in non-pathological cognitive ability measured at ∼83 years after controlling for childhood IQ in the Lothian Birth Cohort 1921 (LBC1921).^4^ Conversely, Taylor *et al*^3^ found little evidence to support an association between retinal fractal dimension and cognitive ability in a sample of 648 LBC1936 participants aged 73 years, before or after adjustment for childhood IQ.

A study of retinal vascular abnormalities in preclinical and clinical Alzheimer's disease found differences between Alzheimer's disease (n=25) and healthy control groups (n=123) for 13 of 19 parameters assessed.^8^ Controls with high neocortical plaque burden (n=15), predictive of progression to dementia,^17^ had increased arteriolar length-to-diameter ratio (LDRa; a measure of vessel width) and venular branching asymmetry factor values. This suggests that LDR may be more sensitive to early vessel morphology changes than summary calibre measures (ie, central retinal arteriolar equivalent, CRAE), where the difference was not observed. To our knowledge, no studies have examined relations between LDR and non-pathological cognitive ageing.

The identification of retinal parameters sensitive to differences in non-pathological cognitive ageing could offer prognostic potential in identifying those at risk of developing mild cognitive impairment or dementia. Here, we examine associations between several retinal vascular parameters and important domains of cognitive ability in a large, well-characterised sample of healthy older adults. IQ scores from age 11 and at age 70 allow us to: investigate the link between retinal measures and childhood cognitive ability; and test associations between retinal measures and lifetime cognitive change by adjusting the cross-sectional retinal–cognitive associations in older age for childhood IQ.

## Methods

### Participants

Participants were from the LBC1936. The recruitment and testing of this cohort have been described elsewhere;^18^ most of the LBC1936 participated in the Scottish Mental Survey 1947 (SMS1947), which tested the intelligence of almost all Scottish schoolchildren born in 1936. Older age data for the present study, including digital retinal photographs and most cognitive tests, were obtained at the second wave of testing (2008–2010) when the participants were ∼73 years old (n=866). A total of 689 participants had at least one image for analysis. Participants with a Mini-Mental State Examination (MMSE) score <24 (n=6) were excluded. This commonly used cut-off reduces the risk of having persons with dementia in the sample.

Ethics approval for the LBC1936 study protocol was obtained from the MultiCentre Research Ethics Committee for Scotland (Wave 1: MREC/01/0/56), the Lothian Research Ethics Committee for Scotland (Wave 1: LREC/2003/2/29) and the Scotland A Research Ethics Committee (Wave 2: 07/MRE00/58). The study complied with the Helsinki Declaration.

### Cognitive ability

#### Moray House Test

The Moray House Test (MHT) No. 12 was used to measure mental ability at ∼11 years as part of the SMS1947.^19^ MHT scores for all LBC1936 participants were corrected for age in days at time of testing and converted to an IQ-type scale (mean=100, SD=15) to provide a measure of age-11 IQ.

#### Change in IQ across the life course

The same version of the MHT was re-administered when participants were seen again at around age 70, and IQ-type scores were calculated using the same method as for age-11 IQ. Age-70 IQ was regressed on age-11 IQ with standardised residuals saved as a measure of IQ change across the life course.

#### Cognitive domains

Factor scores for the domains of general cognitive ability (*g*), processing speed, visuospatial ability, memory and crystallised ability at age 73 were derived from principal component analysis of subtests, according to previous studies of the LBC1936 cohort.^20^ First unrotated principal components explained between 35.4% and 74.7% of the variance in each domain. Factor loadings ranged between 0.44 and 0.94 (see online supplementary table S1 for factor loadings and details of the tests from which factors were derived).

10.1136/bjophthalmol-2016-309017.supp1 supplementary tables

### Retinal image analysis

Digital fundus retinal images were captured at wave 2 of the study at age ∼73 using a non-mydriatic camera at 45° field of view (CRDGi; Canon USA, Lake Success, New York, USA). Images were analysed by a trained grader (MK) at the Centre for Population Health Sciences, University of Edinburgh, using semiautomated software (Singapore I Vessel Assessment (SIVA), V.3, National University of Singapore, Singapore). For each of the 683 participants who had retinal images for analysis, retinal parameters from one eye were measured. The right eye was selected if both images were of the same quality (n=353), or if unavailable or ungradable, the left eye was used (n=330). The main reasons for rejection included images being centred overly towards the macula instead of centred between the optic disc and macula, images being of very poor quality including out-of-focus images and overexposing of the lens, and those with known pathologies including cataract and asteroid hyalosis. Fifteen parameters measured from the calibre (central retinal arteriolar/venular equivalent (CRAE/CRVE); arteriole–venular ratio (AVR); LDR); branching pattern of vessels (number of arterioles and venules with a first branch in Zone C; branching coefficient; junctional exponent deviation) as well as measures of tortuosity and fractal dimension were calculated. Retinal measurement and summarisation procedures have been described previously.^8^ Intragrader and intergrader correlation coefficients are not available for images analysed in the current study. However, intergrader and intraobserver reliability estimates conducted by the same grader as the current study (MK) on sets of 60 images from the Singapore Research Eye Study and Orkney Complex Disease Study confirmed high reliability of quantitative retinal vessel traits (all intraclass correlation coefficients (ICCs) between 0.90 and 0.99).^21^ A description of all retinal parameters is listed in table 1. Separate arteriolar and venular measurements are indicated by lowercase ‘a’ or ‘v’.

**Table 1:** Description of the 15 retinal vascular parameters measured for each retinal photograph and relevant retinal zone

Parameter	Description		Retinal zone
CRAE	Central retinal arteriolar equivalent calibre	Summary measures of vascular equivalent calibre representing the equivalent single-vessel parent width for the six largest arterioles and venules. Based on the Knudston-Parr-Hubbard formula.^24^	B
CRVE	Central retinal venular equivalent calibre		B
AVR	Arteriole–venular ratio	Defined as the ratio of CRAE to CRVE. CRAE/CRVE=AVR	B
FDa	Fractal dimension of arteriolar network	Global summary measure of branching complexity of the retinal vascular tree reflecting how thoroughly the branching pattern fills two-dimensional spaces. Larger values represent a more complex pattern. Calculated from the skeletonised line tracing using the box-counting method.	C
FDv	Fractal dimension of venular network		C
TORTa	Curvature tortuosity arteriole	Integral of the curvature squared along the vessel path, normalised by the total path length. Measurements are summarised to represent the average tortuosity of arterioles and venules separately, with smaller values reflecting straighter vessels.	C
TORTv	Curvature tortuosity venule		C
Num1stBa	Number of first branching arterioles	The number of arterioles and venules with a first bifurcation, branch or daughter vessel, in zone C.	C
Num1stBv	Number of first branching venules		C
BCa	Branching coefficient arteriole	Calculated from average Num1st measurements. Branching coefficient reflects the relationship between parent vessels and branches. Defined as the summed square of the mean vessel widths of each branch or daughter vessel divided by the square of the mean width of the parent vessel.	C
BCv	Branching coefficient venule		C
JEa	Junctional exponent deviation for arterioles	Calculated from average Num1st measurements. Junctional exponent deviation reflects the deviation from the optimum ratio of vessel widths at a bifurcation	C
JEv	Junctional exponent deviation for venules		C
LDRa	Length-to-diameter ratio arteriole	Length-to-diameter ratio is the length of the vessel from the midpoint of one bifurcation to the midpoint of the next bifurcation. It is expressed as a ratio to the diameter of the parent vessel at the first bifurcation	C
LDRv	Length-to- diameter ratio venule		C

Retinal vessels within the retinal zones of interest (0.5–1.0 (zone B) and 1.0–2.0 (zone C) disc diameters away from the optic disc margin) were traced automatically by the software.

### Covariates

Age (in days at time of testing) at the LBC1936 wave 2 testing visit and sex were included as covariates. Smoking status (current, ex or never), apolipoprotein E (*APOE*) status (e4 allele carrier or not), logMAR (logarithm of the minimum angle of resolution) visual acuity, current depression symptoms (depression subscale of Hospital Anxiety and Depression Scale)^22^ and self-reported medical history (classified according to whether they had histories of hypertension, cardiovascular disease (CVD), stroke and diabetes) were ascertained during structured interviews with a trained psychologist and during physical assessment with a research nurse at wave 2 cognitive testing (age ∼73). At Wave 1 (age ∼70), education was recorded as the number of years spent in full-time education, and socioeconomic status (SES) was calculated according to the Office of Population Consensus Surveys (1980).^23^ SES was based on highest status occupation or, for females, husband's occupation if higher. These covariates were selected on the basis of their known association with study outcomes.

### Statistical analysis

Statistical analyses were conducted using SPSS V.19 (IBM, New York, USA) and R V. 2.15.2. Branching coefficient, LDR and tortuosity underwent natural log transformation to normalise their distribution. Remaining variables were normally distributed. Pearson's correlations were used to examine bivariate retinal–cognitive ability associations at age ∼73 (see online supplementary table S2). Multivariate associations were examined using linear regression, with retinal vascular predictors (treated as continuous variables), cognitive ability outcomes and adjustments for potential confounding factors added cumulatively. Model 1 and all subsequent models adjusted for age and sex. Model 2 added hypertension, diabetes, CVD, stroke, smoking status, *APOE* status, current depression symptoms and visual acuity of both eyes. Model 3 further adjusted for SES, education and age-11 IQ. Data are presented as standardised β coefficients (β) reflecting change in cognitive domain score associated with an increase of 1 SD unit in retinal parameter. To minimise the potential for type I errors, p values were adjusted according to the false discovery rate (FDR) method.^25^

Since associations might be confounded by the presence of diabetes or CVD,^2^ we conducted sensitivity analyses excluding individuals with these conditions (diabetes: n=62; CVD: n=189). Changes to results were minimal (see online supplementary tables S5 and S6).

## Results

The sample comprised 683 (51.5% male) participants who underwent cognitive testing at ∼73 years (m=72.5, SD=0.7) and had at least one retinal vascular measurement recorded at the same visit. Demographic characteristics are presented in table 2. Retinal vascular values are summarised in online supplementary table S3.

### Bivariate analyses

Few retinal vascular parameters were associated with cognitive ability at age ∼73, age-11 IQ or with IQ change across the life course (see online supplementary table S1). Venular tortuosity (TORTv) was negatively associated with memory (r=−0.08, p=0.04), crystallised ability (r*=−*0.09, p=0.02) and age-11 IQ (r=−0.10, p=0.02). Arteriolar fractal dimension (FDa) was negatively associated with memory (r=−0.09, p=0.03), and venular fractal dimension (FDv) was negatively associated with age-11 IQ (r=−0.08, p=0.04). IQ change across the life course was negatively associated with arteriolar junctional exponent (JEa; r=−0.11, p=0.01) and positively associated with arteriolar branching coefficient (BCa; r=0.10, p=0.01). These bivariate associations were not corrected for multiple testing. None would have survived FDR correction, which was applied to the regression analyses that follow.

**Table 2:** Characteristics of the study population (% prevalence and mean values)

Participant characteristics	N (%)/M (SD)	Min	Max
Age	72.5 (0.7)	70.9	74.1
Sex			
Male	352 (51.5)		
Female	331 (48.5)		
Presence or history of disease			
Hypertension	322 (47.1)		
Diabetes	62 (9.1)		
CVD	189 (27.7)		
Stroke	38 (5.6)		
Smoking status			
Current smoker	54 (7.9)		
Ex-smoker	308 (45.1)		
Never smoked	321 (47.0)		
*APOE* status			
e4 allele present	184 (26.9)		
No e4 allele	465 (68.1)		
Visual acuity (left)	0.4 (0.3)	−0.1	1.3
Visual acuity (right)	0.4 (0.3)	−0.1	1.4
HADS depression	2.5 (2.1)	0	13
Years of education	10.8 (1.1)	9	14
Social class	2.3 (0.9)		
I	134 (19.6)		
II	261 (38.2)		
IIIN	142 (20.8)		
IIIM	109 (16.0)		
IV	22 (3.2)		
V	5 (0.7)		

Visual acuity in logMAR units. Social classes are categorised as follows: I (professional occupations); II (managerial and technical occupations); IIIN (non-manual skilled occupations); IIIM (manual skilled occupations); IV (partly skilled occupations); V (unskilled occupations).

*APOE,* apolipoprotein E; CVD, cardiovascular disease; HADS, Hospital Anxiety and Depression Scale.

### Regression analyses

The results of multiple linear regressions are presented in table 3. In model 1 which adjusted for age and sex, higher LDRv, FDa and TORTv were nominally significantly associated with poorer speed (LDRv: β=−0.112, p=0.04), memory (FDa: β=−0.087, p=0.04; TORTv: β=−0.085, p=0.04) and crystallised ability (TORTv: β=−0.093, p=0.02), respectively. Increased BCa and decreased JEa were nominally significantly associated with less decline in IQ in old age (BCa: β=0.104, p=0.01; JEa: β=−0.116, p=0.01). Model 2 included adjustment for vascular risk factors. Nominal significance was maintained in the above associations except that between FDa and memory. In model 3, with additional adjustment for SES, education and age-11 IQ, the following associations remained nominally significant: LDRv and speed (β=−0.116, p=0.01), JEa, BCa and IQ change (JEa: β=−0.101, p=0.02; BCa: β=0.089, p=0.04).Two associations became nominally significant after full adjustment. LDRv became negatively associated with *g* (β=−0.079, p=0.04), and increased LDRa became associated with better visuospatial ability (β=0.092, p=0.04). Collinearity in the data was assessed with variance inflation factor (VIF) and tolerance statistics which were found to be within acceptable boundaries in all models (VIF<10; tolerance >1, <3).

**Table 3:** Standardised coefficients from multiple linear regressions using retinal vascular parameters to predict cognitive ability in the LBC1936

		Model 1		Model 2		Model 3				Model 1		Model 2		Model 3	
Cognitive outcome	Retinal predictor	β‡	p Value	β‡	p Value	β‡	p Value	Cognitive outcome	Retinal predictor	β‡	p Value	β‡	p Value	β‡	p Value
*g*								Memory							
	CRAE	−0.003	0.95	0.012	0.79	−0.001	0.97		CRAE	−0.007	0.87	−0.006	0.88	−0.014	0.71
	CRVE	0.010	0.81	0.020	0.63	0.010	0.74		CRVE	0.030	0.47	0.024	0.57	0.022	0.55
	AVR	−0.017	0.69	−0.013	0.76	−0.013	0.67		AVR	−0.040	0.35	−0.033	0.44	−0.036	0.34
	JEa	−0.048	0.26	−0.056	0.20	−0.026	0.38		JEa	−0.030	0.48	−0.035	0.42	−0.019	0.62
	JEv	0.001	0.98	−0.006	0.89	−0.035	0.25		JEv	0.029	0.50	0.030	0.48	0.014	0.71
	FDa	−0.028	0.51	−0.007	0.88	0.014	0.65		FDa	−0.087	0.04*	−0.080	0.06†	−0.062	0.09†
	FDv	−0.038	0.37	−0.023	0.58	0.016	0.61		FDv	−0.020	0.64	−0.013	0.77	0.019	0.61
	TORTa	0.016	0.70	0.025	0.56	0.014	0.64		TORTa	−0.040	0.34	−0.041	0.33	−0.041	0.27
	TORTv	−0.075	0.07†	−0.067	0.12	0.002	0.96		TORTv	−0.085	0.04*	−0.095	0.02*	−0.053	0.16
	LDRa	0.014	0.80	0.006	0.92	0.033	0.38		LDRa	0.013	0.81	0.002	0.96	0.011	0.81
	LDRv	−0.071	0.20	−0.071	0.20	−0.079	0.04*		LDRv	−0.008	0.88	−0.007	0.90	−0.014	0.78
	BCa	0.060	0.16	0.063	0.14	0.037	0.22		BCa	0.050	0.25	0.054	0.21	0.038	0.32
	BCv	−0.033	0.44	−0.025	0.57	−0.016	0.59		BCv	−0.043	0.31	−0.039	0.36	−0.034	0.36
	Num1stBa	−0.047	0.26	−0.039	0.35	−0.040	0.18		Num1stBa	−0.060	0.15	−0.060	0.16	−0.062	0.09†
	Num1stBv	−0.009	0.84	0.002	0.96	−0.013	0.65		Num1stBv	0.019	0.65	0.024	0.57	0.017	0.65
Visuospatial								Crystallised							
	CRAE	−0.019	0.64	−0.002	0.95	−0.008	0.83		CRAE	0.003	0.94	0.002	0.96	0.007	0.81
	CRVE	0.021	0.61	0.031	0.45	0.031	0.39		CRVE	−0.011	0.78	−0.009	0.84	−0.007	0.82
	AVR	−0.048	0.25	−0.040	0.33	−0.043	0.24		AVR	0.008	0.84	0.005	0.91	0.012	0.68
	JEa	−0.052	0.22	−0.059	0.15	−0.040	0.27		JEa	−0.041	0.33	−0.043	0.31	−0.009	0.77
	JEv	0.007	0.86	−0.002	0.95	−0.021	0.57		JEv	0.007	0.86	0.007	0.86	−0.022	0.45
	FDa	−0.008	0.85	0.015	0.71	0.033	0.36		FDa	−0.028	0.50	−0.018	0.67	0.014	0.62
	FDv	−0.019	0.65	0.001	0.98	0.027	0.46		FDv	−0.025	0.55	−0.023	0.59	0.020	0.50
	TORTa	0.021	0.60	0.034	0.40	0.028	0.44		TORTa	0.021	0.62	0.020	0.63	0.014	0.62
	TORTv	−0.032	0.44	−0.018	0.66	0.032	0.38		TORTv	−0.093	0.02*	−0.088	0.04*	−0.011	0.70
	LDRa	0.077	0.14	0.073	0.16	0.092	0.04*		LDRa	−0.027	0.60	−0.033	0.53	−0.012	0.76
	LDRv	−0.046	0.39	−0.045	0.40	−0.053	0.25		LDRv	0.026	0.62	0.030	0.59	0.022	0.56
	BCa	0.070	0.09†	0.073	0.08†	0.056	0.12		BCa	0.032	0.46	0.032	0.45	−0.001	0.97
	BCv	−0.059	0.16	−0.046	0.26	−0.043	0.23		BCv	0.002	0.97	0.002	0.95	0.012	0.69
	Num1stBa	−0.010	0.81	0.004	0.92	0.005	0.89		Num1stBa	−0.052	0.21	−0.046	0.27	−0.044	0.12
	Num1stBv	0.006	0.89	0.014	0.73	0.003	0.92		Num1stBv	−0.004	0.92	0.002	0.97	−0.006	0.84
Speed								IQ change							
	CRAE	−0.024	0.56	−0.002	0.97	−0.009	0.81		CRAE	−0.048	0.25	−0.049	0.25	−0.040	0.35
	CRVE	−0.046	0.27	0.030	0.47	−0.033	0.38		CRVE	−0.052	0.22	−0.050	0.24	−0.047	0.27
	AVR	0.021	0.62	0.027	0.52	0.025	0.51		AVR	−0.003	0.94	−0.006	0.89	0.001	0.98
	JEa	−0.033	0.43	−0.043	0.30	−0.026	0.48		JEa	−0.116	0.01*	−0.112	0.01*	−0.101	0.02*
	JEv	0.011	0.80	−0.002	0.96	−0.020	0.59		JEv	−0.069	0.10	−0.069	0.10	−0.073	0.08†
	FDa	0.036	0.39	0.055	0.19	0.070	0.06†		FDa	−0.032	0.44	−0.033	0.44	−0.030	0.48
	FDv	−0.028	0.51	−0.014	0.74	0.014	0.71		FDv	−0.047	0.27	−0.042	0.33	−0.044	0.30
	TORTa	0.013	0.76	0.020	0.62	0.012	0.75		TORTa	−0.017	0.68	−0.012	0.77	−0.023	0.59
	TORTv	−0.037	0.38	−0.025	0.55	0.022	0.56		TORTv	−0.044	0.30	−0.043	0.32	−0.026	0.54
	LDRa	0.014	0.80	0.004	0.95	0.015	0.76		LDRa	−0.048	0.37	−0.050	0.36	−0.042	0.44
	LDRv	−0.112	0.04*	−0.115	0.03*	−0.116	0.01*		LDRv	−0.008	0.89	−0.017	0.76	−0.007	0.91
	BCa	0.034	0.42	0.043	0.32	0.028	0.46		BCa	0.104	0.01*	0.099	0.02*	0.089	0.04*
	BCv	−0.042	0.32	−0.032	0.44	−0.029	0.43		BCv	0.048	0.26	0.048	0.25	0.046	0.27
	Num1stBa	−0.030	0.47	−0.020	0.63	−0.020	0.58		Num1stBa	−0.052	0.21	−0.058	0.17	−0.053	0.21
	Num1stBv	−0.036	0.39	−0.027	0.51	−0.033	0.37		Num1stBv	−0.048	0.25	−0.051	0.23	−0.052	0.21

Negative associations were such that increased retinal vascular measurements were related to lower cognitive ability scores at age 73 and lower relative change in IQ. Positive associations showed that increased retinal vascular measurements were related to higher cognitive ability scores at age 73 and increased IQ after age 11 or less decline in older age. Model 1 adjusted for age and sex; Model 2 adjusted for age, sex, hypertension, diabetes, cardiovascular history, stroke, current smoking status, apolipoprotein E (*APOE*) status, visual acuity, depression; Model 3 adjusted for age, sex, hypertension, diabetes, cardiovascular history, stroke, current smoking status, *APOE* status, visual acuity, depression, age-11 IQ, years of education and socioeconomic status. No associations survived false discovery rate (FDR) adjustment. N varies due to incomplete range of measurements/missing subtest data: range 553–570 for all retinal parameters except LDR, range: 334–351.

*Significant (p<0.05); **Significant (p<0.01); †Trend (p<0.01).

‡Data are presented as standardised β coefficients (β) reflecting change in cognitive domain score associated with an increase of 1 SD unit in retinal parameters.

Crystallised, crystallised ability; *g*, general cognitive ability; memory, memory ability; speed, information-processing speed; visuospatial, visuospatial ability.

AVR, arteriole–venular ratio; BCa, arteriolar branching coefficient; BCv, venular branching coefficient; CRAE, central retinal arteriolar equivalent; CRVE, central retinal venular equivalent; FDa, arteriolar fractal dimension; FDv, venular fractal dimension; JEa, arteriolar junctional exponent; JEv, venular junctional exponent; LDRa, arteriolar length-to-diameter ratio; LDRv, venular length-to-diameter ratio; Num1stBa, number of first branching arterioles; Num1stBv, number of first branching venules; TORTa, arteriolar tortuosity TORTv, venular tortuosity.

In summary, retinal parameters contributed <1% of the variance in the majority of associations observed. A small number of associations remained significant after adjustment for additional covariates in models 2 and 3. However, no nominally significant associations remained significant following FDR adjustment. Full results are presented in online supplementary table S4.

To check whether our non-significant results were due to a lack of statistical power, we conducted post hoc power analyses. Power calculations were carried out on all retinal parameters. The full sample size (n=570) had 80% power to detect a change of 0.118 SD units. The reduced sample (n=334) had 80% power to detect a change of 0.154 SD units (from http://hedwig.mgh.harvard.edu/sample_size/size.html).

## Discussion

The retinal vessels provide a unique insight into the cerebral microvasculature due to the anatomical, physiological and developmental homology between the retina and brain.^2^ Therefore, this study investigated the contribution of retinal features to non-pathological cognitive ageing. Few retinal–cognitive associations were found, despite the large N and comprehensive assessment of cognitive domains and retinal vessel parameters. After adjustment for childhood IQ and cardiovascular risk factors, decreased arteriolar junctional exponent and increased arteriolar branching coefficient values were significantly associated with less cognitive decline across the life course. Associations between venular LDR, and *g* and processing speed were also maintained. However, all significant associations were small, and none survived FDR adjustment.

The lack of association between cognitive ability and summary calibre measures (CRAE, CRVE, AVR) follows previous findings.^4^ ^6^ Associations between vessel width and dementia^7–9^ suggest that alterations may not manifest until late in the disease process, or that alternative measurements such as LDR may be more sensitive. Only Frost *et al*^8^ corrected for multiple statistical testing. Though the association between increased LDR and slower processing speed did not survive FDR adjustment, it provides tentative support for this idea and should be the subject of further research.

Results from a previous LBC1936 study found little evidence of an association between fractal dimension and cognitive ability.^3^ Though an association between higher fractal dimension and poorer memory performance was found, the low number of significant associations prevents type 1 error being ruled out. This conflicts with studies reporting lower fractal dimensions in those with cognitive dysfunction and dementia.^7–9^ There are therefore inconsistencies in the direction of association between those with cognitive impairment and ‘normal’ cognitive ageing, which further studies might resolve.

This study has some strengths. Using data from a well-characterised cohort, we were able to control for premorbid cognitive ability and numerous possible confounders to provide a more accurate reflection of any independent contribution of retinal properties to cognitive ability in later life. Having childhood IQ meant that we could protect against confounding and/or reverse causation, whereby those with higher cognitive scores in early life have more optimal retinal vessel parameters in older age.^15^ The sample's narrow age range is valuable as it avoids problems associated with heterogeneity of the effects of retinal properties on cognitive function at different ages. Post hoc power calculations suggest that the sample was large enough to detect small effects, and is unlikely to have resulted in type 2 statistical errors. A number of important cognitive domains were assessed, each of which was the reliable summary of multiple tests.

Limitations should be noted. Despite retinal measurement using a validated program (SIVA), there remains a degree of subjective human interaction with potential intergrader and intragrader variability. Retinal vessel calibres are also subject to variation within an individual: arteriolar and venular calibres can vary by up to 17% and 11%, respectively, due to vasomotion and pulsation during a cardiac cycle.^26^ Capturing images at random points during the cycle potentially introduces variation in measurements.

Findings are based on assessment of the best quality image (either right or left) for each participant. This relies on the assumption of symmetry, that is, measurements of retinal parameters from one eye can adequately represent the same parameter in the other eye. Substantial correlations between right and left eye vessel widths and tortuosity have been reported^27^ ^28^ however, only moderate correlations between fractal dimension of each eye were reported in a previous study of LBC1936 participants.^3^

In summary, despite a relatively large sample (with power to detect small effect sizes) and the use of multiple retinal variables and key cognitive domains, no FDR-corrected significant associations between retinal vascular properties and cognitive ability were found. This supports previous negative results concerning milder retinal vascular changes.^3^ ^4^ Associations might have been found, had retinal measurements been available from participants in middle-age, where stronger associations have been reported.^11^ As brain structure and cognitive ability undergo rapid change after age 70,^29^ follow-up retinal photography could provide a better opportunity to assess the relationship between retinal parameters and cognitive change with the progression of vascular pathology. Though mostly null, we judge the present study's results to be valuable. There is currently great interest in finding early and easily accessible biomarkers of age-related cognitive decline. Here, in a large sample with comprehensive cognitive testing, little age variation, multiple retinal parameters, many relevant covariates and rarely available prior cognitive ability, we find that in non-pathological cognitive decline, retinal information, at best, has a very small contribution to make in discriminating who is cognitively ageing better or worse.

## References

[1] O'Brien JT, Erkinjuntti T, Reisberg B, et al Vascular cognitive impairment. Lancet Neurol 2003;2:89–98. 10.1016/S0140-6736(15)00463-8 12849265

[2] Patton N, Aslam T, Macgillivray T, et al Retinal vascular image analysis as a potential screening tool for cerebrovascular disease: a rationale based on homology between cerebral and retinal microvasculatures. J Anat 2005;206:319–48. 10.1111/j.1469-7580.2005.00395.x 15817102 PMC1571489

[3] Taylor AM, MacGillivray TJ, Henderson RD, et al Retinal vascular fractal dimension, childhood IQ, and cognitive ability in old age: the Lothian birth cohort study 1936. PloS ONE 2015;10:e0121119 10.1371/journal.pone.0121119 25816017 PMC4376388

[4] Patton N, Pattie A, MacGillivray T, et al The association between retinal vascular network geometry and cognitive ability in an elderly population. Invest Ophthalmol Vis Sci 2007;48:1995–2000. 10.1167/iovs.06-1123 17460252

[5] Liew G, Mitchell P, Wong TY, et al Retinal microvascular signs and cognitive impairment. J Am Geriatr Soc 2009;57:1892–6. 10.1111/j.1532-5415.2009.02459.x 19737331

[6] Wong TY, Klein R, Sharrett AR, et al Retinal microvascular abnormalities and cognitive impairment in middle-aged persons: the atherosclerosis risk in communities study. Stroke 2002;33:1487–92. 10.1161/01.STR.0000016789.56668.43 12052979

[7] Cheung CYL, Ong YT, Ikram MK, et al Microvascular network alterations in the retina of patients with Alzheimer's disease. Alzheimers Dement 2014;10:135–42. 10.1016/j.jalz.2013.06.009 24439169

[8] Frost S, Kanagasingam Y, Sohrabi H, et al Retinal vascular biomarkers for early detection and monitoring of Alzheimer's disease. Transl Psychiatry 2013;3:e233 10.1038/tp.2012.150 23443359 PMC3591002

[9] Williams MA, McGowan AJ, Cardwell CR, et al Retinal microvascular network attenuation in Alzheimer's disease. Alzheimers Dement (Amst) 2015;16:229–35. 10.1016/j.dadm.2015.04.001 PMC4629099 26634224

[10] Ding J, Patton N, Deary IJ, et al Retinal microvascular abnormalities and cognitive -dysfunction: a systematic review. Br J Ophthalmol 2008;92:1017–25. 10.1136/bjo.2008.141994 18614569

[11] Baker ML, Larsen EM, Kuller LH, et al Retinal microvascular signs, cognitive function, and dementia in older persons. Stroke 2007;38:2041–7. 10.1161/STROKEAHA.107.483586 17525385

[12] Ferguson SC, Blane A, Perros P, et al Cognitive ability and brain structure in Type 1 diabetes: relation to microangiopathy and preceding severe hypoglycemia. Diabetes 2003;52:149–56. 10.2337/diabetes.52.1.149 12502506

[13] Heringa SM, Bouvy WH, van den Berg E, et al Associations between retinal microvascular changes and dementia, cognitive functioning, and brain imaging abnormalities: a systematic review. J Cereb Blood Flow Metab 2013;33:983–95. 10.1038/jcbfm.2013.58 23591648 PMC3705441

[14] Mimoun L, Massin P, Steg G Retinal microvascularisation abnormalities and cardiovascular risk. Arch Cardiovasc Dis 2009;102:449–56. 10.1016/j.acvd.2009.02.008 19520331

[15] Gow AJ, Johnson W, Pattie A, et al Stability and change in intelligence from age 11 to ages 70, 79, and 87: the Lothian birth cohorts of 1921 and 1936. Psychol Aging 2011;26:232–40. 10.1037/a0021072 20973608

[16] Shalev I, Moffitt TE, Wong TY, et al Retinal vessel caliber and lifelong neuropsychological functioning retinal imaging as an investigative tool for cognitive epidemiology. Psychol Sci 2013;24:1198–207. 10.1177/0956797612470959 23678508 PMC3713191

[17] Sperling RA, Aisen PS, Beckett LA, et al Toward defining the preclinical stages of Alzheimer's disease: recommendations from The National Institute on Aging-Alzheimer's Association workgroups on diagnostic guidelines for Alzheimer's disease. Alzheimers Dement 2011;7:280–92. 10.1016/j.jalz.2011.03.003 21514248 PMC3220946

[18] Deary IJ, Gow AJ, Pattie A, et al Cohort profile: the Lothian Birth Cohorts of 1921 and 1936. Int J Epidemiol 2012;41:1576–84. 10.1093/ije/dyr197 22253310

[19] Scottish Council for Research in Education. The intelligence of Scottish children: a National Survey of an age-group. London: University of London Press, 1933.

[20] Tucker-Drob EM, Briley DA, Starr JM, et al Structure and correlates of cognitive aging in a *narrow* age cohort. Psychol Aging 2014;29:236–49. 10.1037/a0036187 24955992 PMC4067230

[21] Kirin M Genetic analysis of retinal traits. 2014.

[22] Zigmond AS, Snaith RP The hospital anxiety and depression scale. Acta Psychiatr Scand 1983;67:261–370. 10.1111/j.1600-0447.1983.tb09716.x 6880820

[23] Office of Population Censuses and Surveys. Classification of occupations 1980. London, UK: Her Majesty's Stationary Office, 1980.

[24] Knudtson MD, Lee KE, Hubbard LD, et al Revised formulas for summarizing retinal vessel diameter. Current Eye Research 2003;27:143–9. 10.1214/aos/1013699998 14562179

[25] Benjamini Y, Yekutieli D The control of the false discovery rate in multiple testing under dependency. Ann Stat 2001;29:1165–88. 10.1214/aos/1013699998

[26] Knudtson MD, Klein BEK, Klein R, et al Variation associated with measurement of retinal vessel diameters at different points in the pulse cycle. Br J Ophthalmol 2004;88:57–61. 10.1136/bjo.88.1.57 14693774 PMC1771926

[27] Leung H, Wang JJ, Rochtchina E, et al Computer-assisted retinal vessel measurement in an older population: correlation between right and left eyes. Clin Experiment Ophthalmol 2011;31:326–30. 10.1046/j.1442-9071.2003.00661.x 12880458

[28] Taarnhøj NCBB, Munch IC, Sander B, et al Straight versus tortuous retinal arteries in relation to blood pressure and genetics. Br J Ophthalmol 2008;92:1055–60. 10.1136/bjo.2007.134593 18653600

[29] Wardlaw JM, Bastin ME, Valdés Hernández MC, et al Brain aging, cognition in youth and old age and vascular disease in the Lothian Birth Cohort 1936: rationale, design and methodology of the imaging protocol. Int J Stroke 2011;6:547–59. 10.1111/j.1747-4949.2011.00683.x 22111801

