# Supplementary material for: Retinal microvascular network geometry and cognitive abilities in community-dwelling older people: The Lothian Birth Cohort 1936 study
Source: Br J Ophthalmol. 2017 Jun 22;101(7):993–8. doi: 10.1136/bjophthalmol-2016-309017 (PMC5530803; doi:10.1136/bjophthalmol-2016-309017)
Supplement: supplementary tables [file bjophthalmol-2016-309017supp001.pdf]

Supplemental Table S1. Domain Score Composition, Factor Loadings from Principal Components Analysis of Cognitive Ability Domains and Cronbach's alpha reliabilities

Domain			Unrotated factor loadings	% variance	Cronbach's α
Visuospatial				53.9	.54
	The visuospatial factor was derived from Matrix Reasoning and Block Design scores from WAIS-III and Spatial Span Forward and Spatial Span Backward from the Wechsler Memory Scale-Third Edition (WMS-III).[1]	Block design	.80		
		Spatial span backward	.72		
		Matrix reasoning	.72		
		Spatial span forward	.69		
Speed				57.7	.56
	The processing speed factor was derived from scores on Symbol Search and Digit Symbol tests from the Wechsler Adult Intelligence Scale-Third Edition (WAIS-III)[2], visual Inspection Time, and 4-Choice Reaction Time mean.[3,4]	Digit symbol coding	.83		
		Symbol search	.81		
		Inspection time	-.77		
		4-Choice reaction time	.61		
Memory				55.7	.54
	The memory factor was derived from Logical Memory, Digit Span Backwards and Verbal Paired Associates from the WMS-III.	Logical Memory	.81		
		Verbal Paired Associates	.80		
		Digit Span Backwards	.62		
Crystallized				74.7	.74
	The crystallized cognitive ability factor was derived from scores on the National Adult Reading Test (NART),[5] the Wechsler Test of Adult Reading (WTAR),[6] and Verbal Fluency.[7]	NART	.94		
		WTAR	.94		
		Verbal Fluency	.70		
G				35.4	.80
	A general cognitive ability factor (*g*) was derived from all 14 subtests on which other domain scores were based.	WTAR	.74		
		NART	.73		
		Digit Symbol coding	.70		
		Symbol search	.70		
		Block design	.65		
		Matrix reasoning	.65		
		Verbal fluency	.58		
		4-Choice reaction time	-.55		
		Digit span backwards	.55		
		Logical memory	.53		
		Verbal paired associates	.49		
		Inspection time	.49		
		Spatial span backward	.47		
		Spatial span forward	.44		

Note. Visuospatial=Visuospatial ability; Speed = information processing speed; Memory = memory ability; Crystallized=Crystallized ability; G=general cognitive ability; WTAR=Wechsler Test of Adult Reading; NART=National Adult Reading Test.

Supplemental Table S2. Correlations between Retinal Vascular Parameters and Cognitive Ability Outcomes

	1.	2.	3.	4.	5.	6.	7.	8.	9.	10.	11.	12.	13.	14.	15.	16.	17.	18.	19.	20.	21.	22.
1. CRAE	-																					
2. CRVE	.51***	-																				
3. AVR	.51***	-.47***	-																			
4. LDRa	-.09	-.01	-.08	-																		
5. LDRv	-.11*	-.10*	-.01	.11	-																	
6. JEa	.24***	.03	.21***	.04	.05	-																
7. JEv	-.04	.19***	.23***	.04	.09	.05	-															
8. FDa	.19***	.09*	.11**	.04	.03	.03	.04	-														
9. FDv	-.03	.10**	-.13**	.10*	.07	.03	-.08	.33***	-													
10. Num1stBa	.14***	.07	.08*	.03	.01	-.06	.07	.50***	.18***	-												
11. Num1stBv	-.01	.13***	-.13***	.004	.09	.05	-.01	.20***	.45***	.22*	-											
12. BCa	-.26***	-.02	-.23***	-.03	-.09	.90***	-.10*	-.08*	-.05	.07	-.08*	-										
13. BCv	.07	-.20***	.27***	-.09	-.07	-.04	-.87***	-.05	.07	-.06	.07	.07	-									
14. TORTa	.10**	.08*	.03	.11*	.05	.01	.04	.28***	.15***	.15***	.15***	-.02	-.04	-								
15. TORTv	-.002	.06	-.06	.05	.09	-.004	-.04	.18***	.29***	.09*	.20***	-.01	.05	.32***	-							
16. Visuo	-.05	.01	-.07	.05	-.04	-.06	.004	.01	.01	-.01	.003	.06	-.06	.04	-.01	-						
17. Speed	-.02	-.07	.05	-.01	-.09	-.02	.01	.04	.01	-.03	-.04	-.01	-.04	.04	-.01	.54***	-					
18. Memory	-.01	.01	-.02	.01	-.02	-.02	.01	-.09*	-.02	-.06	.01	.03	-.03	-.01	-.08*	.38***	.37***	-				
19. Cryst	.001	-.03	.03	-.05	.02	-.01	-.003	.03	-.02	-.07	-.002	-.01	.01	.04	-.09*	.38***	.47***	.53***	-			
20. G	-.02	-.02	-.01	-.01	-.07	-.03	-.01	-.02	-.004	-.05	-.01	.02	-.02	.04	-.05	.76***	.81***	.69***	.78***	-		
21. Age 11 IQ	-.01	-.03	.02	-.03	.02	-.004	.04	-.07	-.08*	-.02	.<001	-.01	-.02	-.01	-.10*	.43***	.45***	.51***	.68***	.69***	-	
22. IQ change	-.04	-.03	-.01	-.01	-.04	-.11**	-.05	-.03	-.04	-.05	-.06	.10*	.03	-.01	-.06	.33***	.28***	.26***	.26***	.37***	.000	-

Note. Visuo=Visuospatial ability; Speed = information processing speed; Memory = memory ability; Cryst=Crystallized ability; G=general cognitive ability. Retinal predictor abbreviations explained in Table 1.

* p<.05. ** p<.01. *** p<.001

Supplemental Table S3. Retinal characteristics of Study Population with Images of Sufficient Quality for Analysis

Retinal parameter	N	Mean (SD)	Min	Max
CRAE	683	130 (12.2)	89.9	179
CRVE	683	188 (17.2)	138	273
AVR	683	0.70 (.06)	0.49	0.89
Length diameter ratio arteriole	418	15.8 (8.65)	1.40	72.3
Length diameter ratio venule	419	13.0 (8.98)	0.87	71.1
Junctional exponent deviation arteriole	666	-0.34 (0.39)	-1.26	0.79
Junctional exponent deviation venule	673	0.02 (0.40)	-0.99	0.83
Fractal dimension arteriole	683	1.17 (0.05)	0.99	1.31
Fractal dimension venules	683	1.14 (0.05)	0.99	1.28
Number of first branching arteriole	683	2.95 (1.22)	0	6
Number of first branching venule	683	2.67 (1.04)	0	6
Branching coefficient arteriole	666	1.42 (0.21)	0.79	2.35
Branching coefficient venule	673	1.23 (0.17)	0.71	2.10
Curvature tortuosity arteriole(x $10^{-5)}$	683	6.79 (1.74)	3.70	16.6
Curvature tortuosity venule (x $10^{-5)}$	683	7.71 (2.17)	3.60	23.8

Note. SD=standard deviation. Retinal predictor abbreviations explained in Table 1.

Supplemental Table S4. Standardised Coefficients from Multiple Linear Regressions using Retinal Vascular Parameters to Predict Cognitive Ability in the LBC1936

		**Model 1**					**Model 2**					**Model 3**					
Cognitive outcome	Retinal predictor	n	β^a	*p*	Adjusted R2	ΔR2	n	β^a	*p*	Adjusted R2	ΔR2	n	β^a	*p*	Adjusted R2	ΔR2	FDR adjusted *p*
G																	
	CRAE	546	-.003	.95	.024	.000	546	.012	.79	.037	.030	546	-.001	.97	.52	.47	.97
	CRVE	546	.010	.81	.024	.000	546	.020	.63	.038	.030	546	.010	.74	.52	.47	.85
	AVR	546	-.017	.69	.024	.000	546	-.013	.76	.037	.029	546	-.013	.67	.52	.47	.84
	JEa	533	-.048	.26	.026	.002	533	-.056	.20	.039	.029	533	-.026	.38	.53	.45	.84
	JEv	539	.001	.98	.021	.000	539	-.006	.89	.034	.029	539	-.035	.25	.52	.48	.84
	FDa	546	-.028	.51	.024	.001	546	-.007	.88	.037	.029	546	.014	.65	.52	.48	.84
	FDv	546	-.038	.37	.025	.001	546	-.023	.58	.038	.029	546	.016	.61	.52	.47	.84
	TORTa	546	.016	.70	.024	.000	546	.025	.56	.038	.030	546	.014	.64	.52	.47	.84
	TORTv	546	-.075	.075†	.029	.006	546	-.067	.12	.042	.028	546	.002	.96	.52	.47	.97
	LDRa	334	.014	.80	.022	.000	334	.006	.92	.045	.049	334	.033	.38	.54	.46	.84
	LDRv	334	-.071	.20	.019	.005	334	-.071	.20	.041	.048	334	-.079	.038*	.54	.49	.57
	BCa	533	.060	.16	.028	.004	533	.063	.14	.040	.028	533	.037	.22	.53	.48	.84
	BCv	539	-.033	.44	.022	.001	533	-.025	.57	.035	.029	533	-.016	.59	.52	.48	.84
	Num1stBa	546	-.047	.26	.026	.002	546	-.039	.35	.039	.029	546	-.040	.18	.52	.47	.84
	Num1stBv	546	-.009	.84	.024	.000	546	.002	.96	.037	.029	546	-.013	.65	.52	.48.	.84
Visuospatial																	
	CRAE	567	-.019	.64	.049	.000	567	-.002	.95	.079	.045	567	-.008	.83	.28	.20	.92
	CRVE	567	.021	.61	.049	.000	567	.031	.45	.080	.046	567	.031	.39	.29	.20	.62
	AVR	567	-.048	25	.051	.002	567	-.040	.33	.081	.045	567	-.043	.24	.9	.20	.62
	JEa	553	-.052	.22	.054	.003	553	-.059	.15	.083	.044	553	-.040	.27	.29	.20	.62
	JEv	559	.007	.86	.046	.000	559	-.002	.95	.075	.044	559	-.021	.57	.28	.20	.71
	FDa	567	-.008	.85	.048	.000	567	.015	.71	.079	.045	567	.033	.36	.29	.21	.62
	FDv	567	-.019	.65	.049	.000	567	.001	.98	.079	.045	567	.027	.46	.28	.21	.62
	TORTa	567	.021	.60	.049	.000	567	.034	.40	.080	.046	567	.028	.44	.28	.20	.62
	TORTv	567	-.032	.44	.049	.001	567	-.018	.66	.080	.045	567	.032	38	.29	.20	.62
	LDRa	348	.077	.14	.056	.006	348	.073	.16	.080	.048	348	.092	.044*	.29	.21	.62
	LDRv	347	-.046	.39	.033	.002	347	-.045	.40	.059	.051	347	-.053	.25	.31	.25	.62
	BCa	553	.070	.095†	.056	.005	553	.073	.077†	.085	.044	553	.056	.12	.29	.20	.62
	BCv	559	-.059	.16	.049	.003	559	-.046	.26	.078	.043	559	-.043	.23	.28	.20	.62
	Num1stBa	567	-.010	.81	.049	.000	567	.004	.92	.079	.045	567	.005	.89	.28	.20	.92
	Num1stBv	567	.006	.89	.048	.000	567	.014	.73	.079	.045	567	.003	.92	.28	.20	.92
Speed																	
	CRAE	559	-.024	.56	.025	.001	559	-.002	.97	.051	.041	559	-.009	.81	.25	.20	.81
	CRVE	559	-.046	.27	.027	.002	559	.030	.47	.052	.040	559	-.033	.38	.25	.20	.81
	AVR	559	.021	.62	.025	.000	559	.027	.52	.052	.042	559	.025	.51	.25	.20	.81
	JEa	546	-.033	.43	.027	.001	546	-.043	.30	.052	.040	546	-.026	.48	.26	.21	.81
	JEv	552	.011	.80	.022	.000	552	-.002	.96	.047	.040	552	-.020	.59	.25	.21	.81
	FDa	559	.036	.39	.026	.001	559	.055	.19	.054	.043	559	.070	.060†	.26	.20	.45
	FDv	559	-.028	.51	.025	.001	559	-.014	.74	.051	.041	559	.014	.71	.25	.20	.81
	TORTa	559	.013	.76	.025	.000	559	.020	.62	.052	.042	559	.012	.75	.25	.20	.81
	TORTv	559	-.037	.38	.026	.001	559	-.025	.55	.052	.041	559	.022	.56	.25	.20	.81
	LDRa	345	.014	.80	.019	.000	345	.004	.95	.053	.059	345	.015	.76	.26	.21	.81
	LDRv	342	-.11	.039*	.021	.012	342	-.12	.032*	.094	.060	342	-.12	.012*	.31	.25	.18
	BCa	546	.034	.42	.027	.001	546	.043	.32	.052	.040	546	.028	.46	.26	.21	.81
	BCv	552	-.042	.32	.023	.002	552	-.032	.44	.048	.040	552	-.029	.43	.25	.20	.81
	Num1stBa	559	-.030	.47	.026	.001	559	-.020	.63	.052	.041	559	-.020	.58	.25	.20	.81
	Num1stBv	559	-.036	.39	.026	.001	559	-.027	.51	.052	.041	559	-.033	.37	.252	.20	.81
Memory																	
	CRAE	558	-.007	.87	.023	.000	558	-.006	.88	.023	.015	558	-.014	.71	.25	.23	.81
	CRVE	558	.030	.47	.024	.001	558	.024	.57	.024	.015	558	.022	.55	.25	.23	.81
	AVR	558	-.040	.35	.025	.002	558	-.033	.44	.024	.015	558	-.036	.34	.25	.23	.77
	JEa	544	-.030	.48	.021	.001	544	-.035	.42	.019	.014	544	-.019	.62	.25	.23	.81
	JEv	550	.029	.50	.023	.001	550	.030	.48	.024	.016	550	.014	.71	.27	.22	.81
	FDa	558	-.087	.039*	.031	.007	558	-.080	.059†	.029	.014	558	-.062	.095†	.25	.22	.71
	FDv	558	-.020	.64	.024	.000	558	-.013	.77	.023	.015	558	.019	.61	.25	.23	.81

	TORTa	558	-.040	.34	.025	.002	558	-.041	.33	.025	.015	558	-.041	.27	.25	.23	.77
	TORTv	558	-.085	.042*	.031	.007	558	-.095	.025*	.032	.017	558	-.053	.16	.253	.22	.77
	LDRa	342	.013	.81	.012	.000	342	.002	.96	.012	.025	342	.011	.81	.25	.24	.81
	LDRv	342	-.008	.88	.015	.000	342	-.007	.90	.017	.028	342	-.014	78	.21	.20	.81
	BCa	544	.050	.25	.022	.002	544	.054	.21	.020	.014	544	.038	.32	.27	.23	.77
	BCv	550	-.043	.31	.024	.002	550	-.039	.36	.024	.016	550	-.034	.36	.25	.23	.77
	Num1stBa	558	-.060	.15	.027	.004	558	-.060	.16	.027	.015	558	-.062	.091†	.25	.23	.71
	Num1stBv	558	.019	.65	.024	.000	558	.024	.57	.024	.016	558	.017	.65	.25	.23	.81
Crystallized																	
	CRAE	570	.003	.94	.026	.000	570	.002	.96	.022	.012	570	.007	.81	.54	.51	.90
	CRVE	570	-.011	.78	.026	.000	570	-.009	.84	.022	.012	570	-.007	.82	.54	.51	.90
	AVR	570	.008	.84	.026	.000	570	.005	.91	.022	.012	570	.012	.68	.54	.51	.90
	JEa	556	-.041	.33	.026	.002	556	-.043	.31	.022	.011	556	-.009	.77	.54	.51	.90
	JEv	562	.007	.86	.025	.000	562	.007	.86	.020	.011	562	-.022	.45	.54	.51	90
	FDa	570	-.028	.50	.027	.001	570	-.018	.67	.023	.011	570	.014	.62	.54	.51	.90
	FDv	570	-.025	.55	.026	.001	570	-.023	.59	.023	.012	570	.020	.50	.54	.51	.90
	TORTa	570	.021	.62	.026	.000	570	.020	.63	.023	.012	570	.014	.62	.54	.51	.90
	TORTv	570	-.093	.025*	.034	.009	570	-.088	.036*	.030	.011	570	-.011	.70	.54	.50	.90
	LDRa	351	-.027	.60	.031	.001	351	-.033	.53	.029	.023	351	-.012	.76	.52	.48	.90
	LDRv	349	.026	.62	.017	.001	349	.030	.59	.000	.009	349	.022	.56	.51	.450	.97
	BCa	556	.032	.46	.025	.001	556	.032	.45	.021	.011	556	-.001	.97	.54	.51	.90
	BCv	562	.002	.97	.025	.000	562	.002	.95	.020	.011	562	.012	.69	.54	.51	.90
	Num1stBa	570	-.052	.21	.029	.003	570	-.046	.27	.025	.011	570	-.044	.12	.54	.51	.90
	Num1stBv	570	-.004	.92	.026	.000	570	.002	.97	.022	.012	570	-.006	.84	.54	.51	.90
IQ change																	
	CRAE	567	-.048	.25	.012	.002	567	-.049	.25	.002	.005	567	-.040	.35	.028	.031	1
	CRVE	567	-.052	.22	.013	.003	567	-.050	.24	.002	.005	567	-.047	.27	.029	.031	.56
	AVR	567	-.003	.94	.010	.000	567	-.006	.89	-.001	.005	567	.001	.98	.026	.032	1
	JEa	554	-.12	.006**	.022	.013	554	-.11	.009**	.011	.005	554	-.10	.017*	.036	.030	.26
	JEv	559	-.069	.10	.015	.005	559	-.069	.10	.004	.006	559	-.073	.083†	.031	.032	.42
	FDa	567	-.032	.44	.011	.001	567	-.033	.44	.000	.005	567	-.030	.48	.027	.031	.71
	FDv	567	-.047	.27	.012	.002	567	-.042	.33	.001	.005	567	-.044	30	.054	.032	.56
	TORTa	567	-.017	.68	.010	.000	567	-.012	.77	.000	.005	567	-.023	.59	.027	.032	.73
	TORTv	567	-.044	.30	.012	.002	567	-.043	.32	.001	.005	567	-.026	.54	.027	.030	.73
	LDRa	350	-.048	.37	.003	.002	350	-.050	.36	-.013	.011	350	-.042	.44	.003	.024	.71
	LDRv	346	-.008	.89	.006	.000	346	-.017	.76	-.011	.010	346	-.007	.91	.024	.043	1
	BCa	554	.10	.015*	.019	.011	554	.099	.022*	.008	.005	554	.089	.036*	.034	.031	.27
	BCv	559	.048	.26	.012	.002	559	.048	.25	.002	.006	559	.046	.27	.028	.031	.56
	Num1stBa	567	-.052	.21	.013	.003	567	-.058	.17	.003	.006	567	-.053	.21	.029	.031	.56
	Num1stBv	567	-.048	.25	.012	.002	567	-.051	.23	.002	.006	567	-.052	.21	.029	.032	.56

Note. Visuospatial=Visuospatial ability; Speed = information processing speed; Memory = memory ability; Crystallized=Crystallized ability; G=general cognitive ability. Retinal predictor abbreviations explained in Table 1. Negative associations were such that increased retinal vascular measurements were related to lower cognitive ability scores at age 73 and lower relative change in IQ. Positive associations showed that increased retinal vascular measurements were related to higher cognitive ability scores at age 73, and increased IQ after age 11 or less decline in older age. Model 1 adjusted for age and sex; Model 2 adjusted for age, sex, hypertension, diabetes, cardiovascular history, stroke, current smoking status, APOE status, visual acuity, depression; Model 3 adjusted for adjusted for age, sex, hypertension, diabetes, cardiovascular history, stroke, current smoking status, APOE status, visual acuity, depression, age 11IQ, years of education and socioeconomic status.

[a] Data are presented as standardized beta coefficients (β) reflecting change in cognitive domain score associated with an increase of 1 SD unit in retinal parameter.

* Significant (p<.05)

**Significant (p<.01)

† Trend (p<.01)

Supplemental Table S5. Standardised Coefficients from Multiple Linear Regressions using Retinal Vascular Parameters to Predict Cognitive Ability in the LBC1936 excluding participants with Diabetes

		Model 1					Model 2					Model 3					
Cognitive outcome	Retinal predictor	n	β^a	*p*	Adjusted R2	ΔR2	n	β^a	*p*	Adjusted R2	ΔR2	n	β^a	*p*	Adjusted R2	ΔR2	FDR adjusted *p*
G																	
	CRAE	497	-.011	.80	.030	.000	497	.011	.82	.040	.026	497	-.004	.91	.52	.47	.91
	CRVE	497	.005	.90	.030	.000	497	.023	.60	.041	.026	497	.016	.61	.52	.47	.83
	AVR	497	-.022	.62	.030	.000	497	-.018	.69	.062	.026	497	-.022	.49	.52	.47	.81
	JEa	485	-.052	.24	.039	.003	485	-.051	.25	.064	.025	485	-.020	.54	.54	.48	.81
	JEv	490	-.006	.89	.027	.000	490	-.010	.82	.037	.026	490	-.031	.33	.52	.47	.81
	FDa	497	-.014	.76	.030	.000	497	.007	.88	.040	.026	497	.024	.45	.52	.47	.81
	FDv	497	-.041	.36	.032	.002	497	-.025	.57	.041	.025	497	.011	.74	.52	.47	.87
	TORTa	497	.013	.77	.030	.000	497	.025	.57	.041	.026	497	.029	.36	.52	.47	.81
	TORTv	497	-.089	.043*	.038	.008	497	.18	.084†	.046	.024	497	.008	.81	.52	.47	.87
	LDRa	304	.031	.58	.030	.001	304	.032	.57	.056	.051	304	.051	.20	.52	.46	.81
	LDRv	297	-.084	.14	.029	.007	297	-.088	.13	.053	.050	297	-.093	.022*	.54	.47	.33
	BCa	485	.066	.14	.035	.004	485	.060	.19	.044	.024	485	.031	.33	.53	.48	.81
	BCv	490	-.041	.36	.029	.002	490	-.036	.43	.038	.025	490	-.027	.39	.52	.47	.81
	Num1stBa	497	.024	.59	.031	.001	497	-.028	.53	.041	.025	497	-.033	.30	.52	.47	.81
	Num1stBv	497	-.011	.80	.030	.000	497	-.004	.93	.040	.026	497	-.009	.76	.52	.47	.87
Visuospatial																	
	CRAE	513	-.028	.53	.056	.001	513	-.005	.92	.091	.049	513	-.011	.78	.28	.19	.83
	CRVE	513	.015	.72	.055	.000	513	.032	.46	.092	.050	513	.035	.36	.28	.19	.68
	AVR	513	-.051	.24	.058	.003	513	-.044	.31	.093	.049	513	-.051	.19	.29	.19	.56
	JEa	500	-.045	.30	.059	.002	500	-.045	.30	.094	.050	500	-.027	.48	.29	.19	.73
	JEv	505	.005	.91	.052	.000	505	-.001	.97	.087	.049	505	-.015	.70	.28	.19	.83
	FDa	513	-.011	.79	.055	.000	513	.014	.74	.091	.050	513	.028	.46	.28	.19	.73
	FDv	513	-.025	.56	.056	.001	513	.000	1.00	.091	.049	513	.022	.57	.28	.19	.77
	TORTa	513	.026	.55	.056	.001	513	.040	.34	.092	.051	513	.042	.26	.29	.19	.56
	TORTv	513	-.034	.43	.056	.001	513	-.015	.73	.091	.049	513	.045	.24	.29	.19	.56
	LDRa	313	.082	.14	.058	.007	313	.083	.13	.095	.060	313	.095	.048*	.29	.20	.56
	LDRv	308	-.055	.33	.036	.003	308	-.058	.30	.082	.069	308	-.067	.17	.31	.23	.56
	BCa	500	.070	.11	.062	.005	500	.063	.15	.096	.049	500	.047	.21	.29	.19	.56
	BCv	505	-.070	.10	.057	.005	505	-.062	.15	.091	.048	505	-.059	.12	.28	.19	.56
	Num1stBa	513	-.001	.98	.055	.000	513	.011	.80	.091	.050	513	.011	.78	.28	.19	.83
	Num1stBv	513	.005	.91	.055	.000	513	.012	.77	.091	.050	513	.008	.83	.28	.19	.83
Speed																	
	CRAE	507	-.016	.72	.035	.000	507	.014	.74	.061	.040	507	.006	.87	.26	.20	.87
	CRVE	507	-.039	.38	.037	.002	507	-.017	.69	.061	.039	507	-.018	.64	.26	.20	.83
	AVR	507	.020	.65	.036	.000	507	.028	.52	.061	.040	507	.023	.55	.26	.20	.83
	JEa	495	-.029	.51	.038	.001	495	-.031	.49	.059	.036	495	-.014	.72	.27	.21	.83
	JEv	500	-.008	.86	.031	.000	500	-.018	.69	.055	.039	500	-.032	.41	.26	.20	.83
	FDa	507	.038	.39	.037	.001	507	.057	.19	.064	.042	507	.069	.073†	.26	.20	.55
	FDv	507	-.057	.19	.038	.003	507	-.042	.34	.062	.038	507	-.016	.69	.26	.20	.83
	TORTa	507	.009	.84	.035	.000	507	.019	.65	.061	.040	507	.021	.59	.26	.20	.83
	TORTv	507	-.066	.13	.039	.004	507	-.051	.25	.063	.038	507	.007	.87	.26	.20	.87
	LDRa	312	.022	.69	.028	.001	507	.025	.65	.061	.057	507	.028	.57	.26	.20	.83
	LDRv	304	-.13	.020*	.038	.018	304	-.14	.014*	.083	.069	304	-.14	.005**	.32	.23	.075†
	BCa	495	.043	.34	.039	.002	495	.041	.35	.060	.036	495	.027	.48	.27	.21	.83
	BCv	500	-.045	.31	.033	.002	500	-.037	.40	.056	.038	500	-.032	.41	.26	.20	.83
	Num1stBa	507	-.019	.67	.035	.000	507	-.013	.77	.061	.040	507	-.015	.70	.26	.20	.83
	Num1stBv	507	-.044	.31	.037	.002	507	-.039	.37	.062	.040	507	-.037	.33	.26	.20	.83
Memory																	
	CRAE	507	.004	.92	.020	.000	507	.007	.87	.012	.008	507	-.001	.98	.25	.23	.98
	CRVE	507	.039	.38	.021	.002	507	.037	.41	.014	.008	507	.040	.30	.25	.23	.87
	AVR	507	-.038	.40	.021	.001	507	-.033	.43	.013	.008	507	-.042	.29	.25	.23	.87
	JEa	494	-.038	.40	.018	.001	494	-.039	.39	.009	.008	494	-.020	.62	.25	.24	.87
	JEv	499	.022	.63	.019	.000	499	.022	.62	.012	.009	499	.012	.76	.24	.24	.93
	FDa	507	-.068	.12	.024	.005	507	-.065	.15	.017	.008	507	-.046	.24	.25	.23	.87

	FDv	507	-.019	.67	.020	.000	507	-.012	.79	.013	.008	507	.019	.64	.25	.23	.87
	TORTa	507	-.034	.43	.021	.001	507	-.034	.44	.014	.008	507	-.023	.56	.25	.23	.87
	TORTv	507	-.071	.11	.025	.005	507	-.080	.076†	.019	.009	507	-.027	.49	.25	.23	.87
	LDRa	310	.042	.46	.010	.002	310	.041	.47	-.004	.012	310	.041	.41	.24	.24	.87
	LDRv	304	.002	.97	.014	.000	304	.001	.98	.004	.016	304	-.004	.94	.19	.19	.98
	BCa	494	.049	.27	.019	.002	494	.050	.26	.010	.008	494	.032	.42	.25	.24	.87
	BCv	499	-.040	.37	.021	.002	499	-.038	.40	.013	.009	499	-.033	.40	.25	.23	.87
	Num1stBa	507	-.054	.22	.023	.003	507	-.057	.20	.016	.008	507	-.061	.12	.25	.23	.87
	Num1stBv	507	.007	.88	.020	.000	507	.008	.86	.012	.008	507	.009	.81	.25	.23	.93
Crystallized																	
	CRAE	516	.002	.96	.023	.000	516	.002	.96	.016	.009	516	.005	.88	.56	.53	.95
	CRVE	516	-.016	.71	.023	.000	516	-.011	.81	.016	.009	516	-.002	.95	.56	.53	.95
	AVR	516	.010	.81	.023	.000	516	.006	.90	.016	.009	516	.003	.91	.56	.53	.95
	JEa	503	-.050	.26	.024	.003	503	-.046	.30	.016	.008	503	-.011	.71	.56	.53	.95
	JEv	508	-.005	.91	.021	.000	508	-.005	.91	.014	.008	508	-.026	.39	.56	.53	.95
	FDa	516	-.022	.61	.023	.001	516	-.016	.72	.016	.009	516	.015	.61	.56	.53	.95
	FDv	516	-.037	.40	.024	.001	516	-.032	.48	.017	.009	516	.008	.79	.56	.53	.95
	TORTa	516	.007	.87	.023	.000	516	.010	.83	.016	.009	516	.018	.54	.56	.53	.95
	TORTv	516	-.11	.008**	.036	.013	516	-.109	.014*	.028	.007	516	-.016	.60	.56	.52	.95
	LDRa	316	-.041	.47	.028	.002	316	-.042	.45	.029	.026	316	-.030	.44	.54	.50	.95
	LDRv	310	.012	.83	.013	.000	310	.013	.82	-.005	.008	310	.006	.88	.52	.51	.95
	BCa	503	.045	.31	.023	.002	503	.040	.38	.015	.008	503	.007	.82	.56	.53	.95
	BCv	508	.011	.80	.021	.000	508	.010	.81	.014	.008	508	.018	.56	.55	.53	.95
	Num1stBa	516	-.048	.27	.025	.002	516	-.047	.29	.018	.009	516	-.044	.14	.56	.53	.95
	Num1stBv	516	-.008	.85	.023	.000	516	-.006	.89	.016	.009	516	-.002	.94	.56	.53	.95
IQ change																	
	CRAE	514	-.046	.30	.013	.002	514	-.043	.34	.003	.005	514	-.035	.43	.033	.035	.79
	CRVE	514	-.035	.43	.012	.001	514	-.033	.46	.002	.005	514	-.031	.47	.033	.036	.79
	AVR	514	-.018	.68	.011	.000	514	-.018	.69	.001	.005	514	-.011	.80	.032	.036	.88
	JEa	501	-.11	.010*	.022	.013	501	-.11	.012*	.012	.006	501	-.10	.020*	.042	.035	.30
	JEv	506	-.059	.18	.014	.003	506	-.057	.20	.003	.005	506	-..059	.18	.034	.035	.79
	FDa	514	-.033	.45	.012	.001	514	-.030	.50	.002	.005	514	-.028	.53	.032	.036	.79
	FDv	514	-.041	.35	.013	.002	514	-.037	.41	.002	.005	514	-.040	.37	.033	.036	.79
	TORTa	514	-.006	.88	.011	.000	514	-.004	.93	.001	.005	514	-.011	.80	.032	.036	.88
	TORTv	514	-.028	.53	.012	.001	514	-.027	.55	.001	.005	514	-.011	.81	.032	.035	.88
	LDRa	315	-.019	.74	.000	.000	315	-.021	.71	-.012	.014	315	-.013	.82	.006	.027	.88
	LDRv	308	.006	.92	.006	.000	308	-.002	.97	-.005	.015	308	.003	.96	.036	.049	.96
	BCa	501	.099	.027*	.018	.010	501	.096	.034*	.009	.006	501	.086	.054†	.039	.036	.41
	BCv	506	.038	.39	.012	.001	506	.038	.40	.002	.005	506	.035	.43	.032	.035	.79
	Num1stBa	514	-.049	.27	.013	.002	514	-.050	.26	.003	.006	514	-.044	.31	.034	.035	.79
	Num1stBv	514	-.045	.31	.013	.002	514	-.043	.32	.003	.005	514	-.046	.29	.034	.036	.79

Note. Visuospatial=Visuospatial ability; Speed = information processing speed; Memory = memory ability; Crystallized=Crystallized ability; G=general cognitive ability. Retinal predictor abbreviations explained in Table 1. Negative associations were such that increased retinal vascular measurements were related to lower cognitive ability scores at age 73 and lower relative change in IQ. Positive associations showed that increased retinal vascular measurements were related to higher cognitive ability scores at age 73, and increased IQ after age 11 or less decline in older age. Model 1 adjusted for age and sex; Model 2 adjusted for age, sex, hypertension, diabetes, cardiovascular history, stroke, current smoking status, APOE status, visual acuity, depression; Model 3 adjusted for adjusted for age, sex, hypertension, diabetes, cardiovascular history, stroke, current smoking status, APOE status, visual acuity, depression, age 11IQ; years of education and socioeconomic status.

[a] Data are presented as standardized beta coefficients (β) reflecting change in cognitive domain score associated with an increase of 1 SD unit in retinal parameter.

* Significant (p<.05)

**Significant (p<.01)

† Trend (p<.10)

Supplemental Table S6. Standardised Coefficients from Multiple Linear Regressions Predicting Cognitive Ability in a subset of LBC1936 Excluding Participants with CVD

		**Model 1**					**Model 2**					**Model 3**					
Cognitive outcome	Retinal predictor	n	β^a	*p*	Adjusted R2	∆R2	n	β^a	*p*	Adjusted R2	∆R2	n	β^a	*p*	Adjusted R2	∆R2	FDR adjusted *p*
G																	
	CRAE	402	.005	.91	.034	.000	402	.019	.71	.053	.038	402	-.025	.46	.56	.49	.81
	CRVE	402	.007	.88	.034	.000	402	.014	.78	.053	.038	402	.012	.77	.57	.49	.83
	AVR	402	-.004	.94	.034	.000	402	.003	.96	.053	.038	402	-.038	.28	.56	.49	.81
	JEa	393	-.042	.40	.039	.002	393	-.039	.44	.055	.035	393	-.023	.50	.56	.50	.81
	JEv	396	.004	.93	.030	.000	396	-.001	.98	.048	.037	396	-.016	.65	.55	.50	.81
	FDa	402	-.055	.26	.037	.003	402	-.037	.46	.054	.036	402	-.025	.46	.56	.49	.81
	FDv	402	-.090	.067†	.042	.008	402	-.076	.13	.059	.035	402	-.020	.56	.56	.49	.81
	TORTa	402	-.023	.64	.035	.001	402	-.004	.93	.053	.037	402	-.010	.77	.56	.49	.83
	TORTv	402	-.11	.020*	.047	.013	402	-.10	.038*	.063	.035	402	-.017	.63	.56	.48	.81
	LDRa	234	-.030	.64	.028	.001	234	-.029	.65	.051	.055	234	.004	.93	.56	.49	.93
	LDRv	246	-.051	.42	.016	.003	246	-.040	.53	.048	.063	246	-.046	.29	.57	.50	.81
	BCa	393	.052	.30	.040	.003	393	.048	.34	.055	.035	393	.035	.31	.56	.50	.81
	BCv	396	-.019	.70	.031	.000	396	-.015	.76	.049	.037	396	-.027	.43	.56	.50	.81
	Num1stBa	402	-.053	.28	.037	.003	402	-.045	.36	.055	.037	402	-.063	.059†	.56	.50	.68
	Num1stBv	402	-.063	.20	.038	.004	402	-.050	.31	.056	.036	402	-.057	.090†	.56	.49	.68
Visuospatial																	
	CRAE	415	-.052	.29	.054	.003	415	-.036	.46	.081	.044	415	-.064	.13	.31	.22	.48
	CRVE	415	.009	.85	.051	.000	415	.015	.76	.080	.046	415	.017	.69	.30	.22	.93
	AVR	415	-.064	.19	.055	.004	415	-.055	.26	.082	.045	415	-.084	.049*	.31	.23	.44
	JEa	406	-.067	.16	.061	.005	406	-.064	.19	.085	.042	406	-.053	.21	.31	.22	.52
	JEv	409	.030	.54	.048	.001	409	.022	.64	.075	.045	409	.007	.87	.30	.22	.93
	FDa	415	-.031	.52	.052	.001	415	-.011	.82	.080	.045	415	-.001	.99	.30	.22	.99
	FDv	415	-.090	.061†	.059	.008	415	-.072	.14	.084	.042	415	-.031	.47	.30	.22	.93
	TORTa	415	-.034	.47	.053	.001	415	-.011	.82	.080	.045	415	-.016	.71	.30	.22	.93
	TORTv	415	-.058	.22	.055	.003	415	-.045	.35	.081	.044	415	.016	.71	.30	.22	.93
	LDRa	242	.013	.83	.053	.000	242	.015	.81	.084	.062	242	.036	.51	.32	.24	.93
	LDRv	255	.010	.87	.028	.000	255	.023	.72	.046	.048	255	.009	.87	.32	.27	.93
	BCa	406	.091	.061†	.064	.008	416	.085	.080†	.088	.041	416	.077	.068†	.31	.22	.44
	BCv	409	-.074	.13	.053	.005	409	-.067	.16	.079	.044	409	-.071	.088†	.30	.22	.44
	Num1stBa	415	-.010	.83	.051	.000	415	.002	.97	.079	.046	415	-.012	.77	.30	.22	.93
	Num1stBv	415	-.059	.22	.055	.003	415	-.046	.33	.082	.044	415	-.052	.21	.31	.22	.52
Speed																	
	CRAE	412	.000	1	.026	.000	412	.017	.72	.061	.053	412	-.012	.79	.29	.23	.98
	CRVE	412	-.062	.21	.029	.004	412	-.050	.30	.063	.052	412	-.045	.28	.29	.23	.89
	AVR	412	.067	.18	.030	.004	412	.071	.15	.065	.053	412	.040	.36	.29	.22	.89
	JEa	403	-.032	.52	.029	.001	403	-.030	.53	.061	.051	403	-.023	.59	.30	.24	.96
	JEv	406	.033	.51	.023	.001	406	.023	.64	.056	.051	406	.013	.76	.29	.23	.98
	FDa	412	.015	.76	.026	.000	412	.031	.53	.061	.054	412	.041	.33	.29	.23	.89
	FDv	412	-.061	.21	.029	.004	412	-.050	.31	.063	.052	412	-.004	.93	.29	.23	1
	TORTa	412	-.012	.81	.026	.000	412	.008	.86	.061	.053	412	.000	1	.29	.23	1
	TORTv	412	-.081	.095†	.032	.007	412	-.065	.18	.065	.050	412	-.002	.97	.29	.22	1
	LDRa	242	-.036	.58	.019	.001	242	-.035	.58	.059	.071	242	-.025	.64	.30	.24	.96
	LDRv	253	-.079	.21	.011	.006	253	-.075	.23	.069	.087	253	-.079	.13	.36	.29	.89
	BCa	403	.026	.59	.029	.001	403	.029	.55	.061	.051	403	.024	.57	.30	.24	.96
	BCv	406	-.045	.36	.024	.002	406	-.042	.39	.057	.051	406	-.051	.23	.29	.23	.89
	Num1stBa	412	-.027	.58	.026	.001	412	-.021	.67	.061	.053	412	-.032	.45	.29	.23	.96
	Num1stBv	412	-.076	.12	.031	.006	412	-.064	.18	.065	.051	412	-.065	.12	.29	.23	.89
Memory																	
	CRAE	408	-.023	.65	.034	.001	408	-.016	.74	.031	.017	408	-.043	.33	.24	.22	.82
	CRVE	408	.020	.65	.033	.000	408	.012	.80	.031	.017	408	.016	.71	.25	.22	.94
	AVR	408	-.049	.33	.035	.002	408	-.035	.49	.032	.016	408	-.064	.15	.25	.22	.57
	JEa	399	-.001	.98	.030	.000	399	-.002	.97	.025	.014	399	.004	.94	.24	.21	.94

	JEv	402	.017	.73	.033	.000	402	.012	.80	.030	.017	402	.003	.94	.24	.21	.94
	FDa	408	-.13	.009**	.049	.016	408	-.12	.017*	.045	.015	408	-.11	.013*	.26	.21	.19
	FDv	408	-.020	.69	.033	.000	408	-.007	.89	.031	.017	408	.033	.45	.25	.22	.84
	TORTa	408	-.079	.11	.039	.006	408	-.079	.11	.037	.017	408	-.078	.074†	.25	.22	.37
	TORTv	408	-.099	.042*	.043	.010	408	-.10	.037*	.042	.018	408	-.055	.21	.25	.21	.62
	LDRa	238	.053	.42	.013	.003	238	.039	.55	.010	.031	238	.050	.39	.24	.22	.84
	LDRv	250	-.029	.64	.024	.001	250	-.013	.84	.020	.028	250	-.007	.90	.19	.17	.94
	BCa	399	.022	.65	.031	.000	399	.020	.69	.025	.014	399	.016	.72	.24	.21	.94
	BCv	402	-.024	.63	.033	.001	402	-.016	.75	.030	.017	402	-.023	.60	.24	.21	.94
	Num1stBa	408	-.092	.060†	.041	.008	408	-.082	.094†	.038	.015	408	-.098	.025*	.26	.22	.19
	Num1stBv	408	-.004	.93	.033	.000	408	.005	.91	.031	.017	408	.008	.85	.25	.22	.94
Crystallized																	
	CRAE	417	.017	.73	.030	.000	417	.021	.67	.020	.010	417	-.007	.84	.55	.52	.97
	CRVE	417	.008	.87	.029	.000	417	.010	.84	.020	.009	417	.020	.54	.55	.52	.86
	AVR	417	.004	.93	.029	.000	417	.006	.91	.020	.009	417	-.029	.39	.55	.52	.86
	JEa	408	-.041	.40	.032	.002	408	-.039	.43	.021	.009	408	-.018	.59	.56	.52	.86
	JEv	411	.003	.96	.028	.000	411	.003	.95	.018	.009	411	-.016	.63	.55	.52	.86
	FDa	417	-.011	.82	.029	.000	417	-.004	.9	.020	.009	417	.019	.56	.55	.52	.86
	FDv	417	-.064	.19	.033	.004	417	-.063	.21	.024	.009	417	.001	.97	.55	.52	.97
	TORTa	417	.017	.73	.030	.000	417	.019	.70	.020	.010	417	.020	.54	.55	.52	.86
	TORTv	417	-.12	.012*	.044	.015	417	-.12	.016*	.034	.008	417	-.027	.43	.55	.51	.86
	LDRa	244	-.058	.36	.035	.003	244	-.059	.36	.027	.025	244	-.038	.39	.54	.50	.86
	LDRv	256	.003	.96	.009	.000	256	.010	.88	-.011	.011	256	-.002	.97	.53	.52	.97
	BCa	408	.022	.66	.030	.000	408	.019	.70	.020	.009	408	.005	.89	.56	.53	.97
	BCv	411	.026	.59	.028	.001	411	.026	.60	.018	.009	411	.018	.59	.55	.52	.86
	Num1stBa	417	-.034	.48	.030	.001	417	-.029	.56	.021	.009	417	-.048	.1	.56	.52	.86
	Num1stBv	417	-.038	.43	.031	.001	417	-.031	.53	.021	.009	417	-.029	.38	.55	.52	.86
IQ change																	
	CRAE	414	-.072	.15	.014	.005	414	-.068	.17	.011	.017	414	-.070	.16	.03	.029	.36
	CRVE	414	-.020	.68	.009	.000	414	-.013	.79	.007	.017	414	-.011	.82	.029	.029	.82
	AVR	414	-.057	.26	.012	.003	414	-.060	.23	.010	.018	414	-.064	.20	.033	.029	.36
	JEa	406	-.11	.035*	.018	.011	406	-.095	.057†	.014	.016	406	-.087	.078†	.036	.028	.27
	JEv	408	-.057	.25	.012	.003	408	-.060	.23	.010	.018	408	-.061	.22	.033	.029	.36
	FDa	414	-.071	.15	.014	.005	414	-.066	.19	.011	.017	414	-.061	.22	.033	.028	.36
	FDv	414	-.11	.023*	.021	.012	414	-.11	.036*	.018	.016	414	-.099	.047*	.039	.027	.27
	TORTa	414	-.089	.069†	.017	.008	414	-.083	.092†	.014	.016	414	-.090	.066†	.037	.030	.27
	TORTv	414	-.062	.21	.012	.004	414	-.053	.28	.010	.017	414	-.030	.55	.030	.027	.63
	LDRa	243	-.059	.37	.001	.003	243	-.066	.32	-.014	.019	243	-.064	.33	.004	.030	.45
	LDRv	253	.039	.54	.004	.001	253	.029	.65	-.014	.014	253	.023	.72	.004	.029	77
	BCa	406	.11	.035*	.018	.011	406	.093	.065†	.014	.016	406	.089	.076†	.069	.028	.27
	BCv	408	.037	.46	.010	.001	408	.037	.46	.008	.017	408	.032	.52	.030	.029	.63
	Num1stBa	414	-.054	.28	.010	.018	414	-.056	.26	.010	.018	414	-.052	.29	.032	.028	.44
	Num1stBv	414	-.072	.14	.014	.005	414	-.074	.14	.012	.018	414	-.083	.091†	.036	.030	.27

Note. Visuospatial=Visuospatial ability; Speed = information processing speed; Memory = memory ability; Crystallized=Crystallized ability; G=general cognitive ability. Retinal predictor abbreviations explained in Table 1. Negative associations were such that increased retinal vascular measurements were related to lower cognitive ability scores at age 73, lower relative change in IQ. Positive associations showed that increased retinal vascular measurements were related to higher cognitive ability scores at age 73, and increased IQ after age 11 or less decline in older age.

Model 1 adjusted for age and sex; Model 2 adjusted for age, sex, hypertension, diabetes, stroke, current smoking status, APOE status, visual acuity, depression; Model 3 adjusted for adjusted for age, sex, hypertension, diabetes, stroke, current smoking status, APOE status, visual acuity, depression, age 11IQ; years of education and socioeconomic status.

[a] Data are presented as standardized beta coefficients (β) reflecting change in cognitive domain score associated with an increase of 1 SD unit in retinal parameter.

* Significant ($p<.05$)

**Significant ($p<.01$)

† Trend ($p<.01$)

Supplementary References

1. Wechsler, D. WMS–IIIUK administration and scoring manual. London, UK: Psychological Corporation 1998
2. Wechsler D. WAIS–IIIUK administration and scoring manual. London, UK: Psychological Corporation 1998
3. Deary IJ, Simonotto E, Meyer M, et al. The functional anatomy of inspection time: An event-related fMRI study. *Neuroimage* 2004;22:1466–1479. doi:10.1016/j.neuroimage.2004.03.047
4. Deary IJ, Der G, Ford G. Reaction times and intelligence differences: A population-based cohort study. *Intelligence* 2001;29:389–399. doi:10.1016/S0160-2896(01)00062-9
5. Nelson HE, Willison J. National Adult Reading Test (NART). London, UK: NferNelson 1991
6. Wechsler, D. Wechsler Test of Adult Reading: WTAR. New York, NY: Psychological Corporation 2001.
7. Lezak, M. Neuropsychological testing. Oxford, UK: Oxford University Press 2004
